# Insect Consciousness

**DOI:** 10.3389/fnbeh.2021.653041

**Published:** 2021-05-21

**Authors:** Morten Overgaard

**Affiliations:** ^1^Aarhus Institute of Advanced Studies, Aarhus University, Aarhus, Denmark; ^2^Center for Functionally Integrative Neuroscience, Aarhus University, Aarhus, Denmark

**Keywords:** consciousness, subjective experience, insects, measures of consciousness, methodology, theories of consciousness

## Abstract

The question of consciousness in other species, not least species very physically different from humans such as insects, is highly challenging for a number of reasons. One reason is that we do not have any available empirical method to answer the question. Another reason is that current theories of consciousness disagree about the relation between physical structure and consciousness, i.e., whether consciousness requires specific, say, neural structures or whether consciousness can be realized in different ways. This article sets out to analyze if and how there could be an empirical and/or a theoretical approach to the topic on the basis of current consciousness research in humans.

## Introduction

Whether and to which degree other species are conscious—especially species very structurally different from humans—are questions that we do not know how to think scientifically about yet. First, answering such questions involves clear and precise measures of consciousness. Furthermore, from a theoretical perspective, answering the question requires for us to understand how consciousness relates to its physical substrate as insects obviously have a very different structural composition. The question has divided waters among researchers to a degree that some papers in highly cited journals claim that the question is already resolved (e.g., Barron and Klein, 2016), while others claim that we do not know anything about it at all (e.g., Key et al., 2016).

“Consciousness” has been associated with several different meanings, including self-awareness, knowledge about objects, and subjective experience. In this context, the term “consciousness” is only intended to refer to the latter meaning. Knowledge, including self-knowledge, denote particular cognitive states which enable specific types of behavior. Arguably, computers and robots “have” knowledge about certain facts and objects in their surroundings enabling, say, navigation or communication. Subjective experience, on the contrary, is not associated with any specific functional definition. In consciousness research, subjective experience is in most cases considered the defining feature of consciousness, so that whenever a mental state has any kind of qualitative feel, it is a conscious state (e.g., Nagel, 1974; Jackson, 1986; Searle, 1990, 1992; Chalmers, 1995, 1996). Thomas Nagel states that something is a conscious state when there is something it is like to be in this particular state (Nagel, 1974). This is suggested as a criterion of demarcation for consciousness, so that for any conscious experience, there is something “it is like” for somebody.

Here, I will focus specifically on this latter, most challenging, question whether insects have subjective experience. The intention will not be to evaluate whether insects are “more or less” conscious than, say, humans or what their “level of consciousness” may be (Overgaard and Overgaard, 2011; Bayne et al., 2016), but if there is any subjective experience at all. The intention is not to present a complete review of all possible approaches to the question, or even a characterization of research in insect cognition. I will discuss whether there is a conceivable *methodological* approach to answer the question of consciousness in insects, and whether there is a conceivable *theoretical* approach based on current methods and theories in consciousness research. The discussion will shed light on some of the foundational issues that go before empirical research in insect consciousness, and at the same time, asking this difficult question may in turn inform the current debates in consciousness research.

## Methodological Approaches

Historically, the attempt to “measure” consciousness has unfolded as a debate between direct and indirect approaches. Direct approaches, at least intuitively, are the most informative as participating experimental subjects here simply report about their own experiences. As subjective reports however have demonstrable limits (e.g., lack of insights into personal bias, memory problems etc.), many scientists have refrained from their use and insisted on the use of objective measures only (e.g., Nisbett and Wilson, 1977; Johansson et al., 2006).

Experiments on consciousness that are based on objective measures—the “indirect” approach—typically involve asking subjects to choose between alternatives, e.g., in forced-choice tasks. Although such methods may stay clear of classical limitations of subjective methods, they are confronted with other problems, which, according to some scientists, are greater. For one thing, objective measures must assume that the “threshold” of giving a correct response is the same as the “threshold” of having a subjective experience of the same content (Fu et al., 2008; Timmermans and Cleeremans, 2015). Furthermore, in order to arrive at any one particular objective method, one must have “calibrated it” to something else in order to know that this particular behavior can be considered a measure of consciousness—and not something else. This would typically involve associating a subjective report with a particular behavior—a process by which one would “import” all the weaknesses related to subjective reports that one tried to avoid in the first place (Overgaard, 2010).

Proponents of the “direct” approach have attempted to develop precise and sensitive scales to capture minor variations in subjective experience, e.g., the Perceptual Awareness Scale and gradual confidence ratings (Ramsøy and Overgaard, 2004; Sandberg and Overgaard, 2015). Although different approaches to this idea disagree about what constitutes the optimal measure (Dienes and Seth, 2010; Timmermans et al., 2010; Szczepanowski et al., 2013), they share the view that a detailed subjective report may be imprecise yet better than an indirect measure.

In recent years, the arsenal of indirect measures have been supplied with what is named “no-report paradigms.” Essentially, all paradigms using objective measures only are without report, so in a certain sense, paradigms labeled “no-report paradigms” have not introduced anything new. Nevertheless, experiments of this kind attempt first to associate a particular objective measure (e.g., a behavior or a brain activation) with conscious experience, and then to apply this measure as a measure of consciousness so that no direct report is needed (e.g., Frässle et al., 2014; Pitts et al., 2014). Such methods intuitively seem to circumvent some of the criticism mentioned above. However, and as mentioned above, the only way one may associate a phenomenon as nystagmus with conscious experience is by the direct use of introspection (to establish the “correlation”) (Overgaard and Fazekas, 2016).

In the case of insect consciousness, it is clear that none of the approaches work. We cannot ask insects about the experiences directly. At the same time, we cannot use indirect measures, not even no-report paradigms, as all of these approaches require us to know of some measure—e.g., a type of brain activity—that is already correlated with subjective measures in order for that measure to be associated with consciousness. Even in humans, such generalizations can be questioned, but obviously they cannot be translated to beings with a nervous system but nothing comparable to a human brain at all. However, even if subjective reports may arguably be the “gold standard” in consciousness research, the lack of report does not necessarily mean a lack of consciousness.

The current arsenal of direct and indirect measures thus seems unable to be generalized to very neurally different individuals—let alone species. A few publications have nevertheless claimed otherwise, e.g., Mogensen and Overgaard (2020) who report interesting electrophysiological data with implications for the understanding of the avian prefrontal “cortex.” For almost 40 years it has been known that such a structure exists (Mogensen and Divac, 1982) and like in mammals there has been a focus on “delay tasks” (Mogensen and Overgaard, 2020). Mogensen and Overgaard (2020) add significantly to the understanding of how the posterodorsolateral neostriatum contributes to the mediation of such tasks. The authors, however, also interpret their single cell recordings to reflect conscious perception– and state that their results demonstrate consciousness in birds. Such an approach presupposes that the neural correlate of consciousness (NCC) was identified as a specific and localized activity. Some studies have indicated that prefrontal structures may be involved in the NCC (e.g., Lau and Rosenthal, 2011)—but that is far from always the case (Overgaard and Mogensen, 2020). Barron and Klein (2016) claim that subjective experience is supported by integrated structures in the midbrain that create a neural simulation of the state of the mobile animal in space. As structures in the insect brain perform analogous functions as in humans, they claim as a consequence that the insect brain also supports a capacity for subjective experience. But just as above, this is based on very strong assumptions and claims that the location of the NCC is already resolved, and that this can be generalized in a very simple way, so that all instances of a system with functional similarities with the human brain must also be conscious in the same way.

Some of NCC models (e.g., Overgaard and Mogensen, 2014; Tononi et al., 2016) operate with an NCC that is focused on computational principles rather than any specific structure. But even if certain brain structures should indeed turn out to be necessary for conscious experience in humans, such structural correlates that have already been mapped onto subjective data cannot be assumed to be generalized across time, context, task, person, and species (Overgaard, 2004, 2010).

## Theoretical Approaches

Whereas, we seem to have no current good method to decide whether non-human creatures have subjective experience, still, it is possible to deduce hypotheses about it from current theories of consciousness. One way to divide the waters in the vast sea of theories about consciousness is whether the theories propose that specific neural regions are necessary requirements for consciousness, or whether they are simply sufficient.

Currently, much debate in the neuroscience of consciousness literature centers around whether the neural correlates of consciousness (NCC) are early (e.g., related to occipital cortex when investigating visual consciousness) or late (e.g., related to prefrontal areas). Both types of NCC candidates are backed by experimental investigations, and proponents of both views present evidence going against the opposite proposal. Although they are typically presented as contrasts, they share the idea of associating consciousness with one particular brain region. The underlying assumption for this association could be the belief that brain regions “are” the functions they are associated with, i.e., that the functions are identical with or can be reduced to them. In the context of this article, “structure-function identity” is used to mean the view that a specific mental states depends on a specific neural structure (e.g., that face perception is identical with fusiform gyrus). Of course, the mere correlation of one or another brain area with consciousness does not imply any specific underlying consciousness theory.

One theory that has typically been associated with the view that consciousness relates to prefrontal areas is Higher Order Thought Theory (HOT). HOT posits that a mental state, such as a sensation, is conscious when it is the intentional object of another (higher-order) mental state. Similarly, reflexive theories hold that a mental state has the property of being conscious by instantiating a special reflexive relation to itself. Both theories are similar in the sense that they both seek to explain consciousness by reference to properties of—or relations between—mental states. HOT—as a theory of consciousness—is not dependent on any particular brain location being the proper NCC, i.e., even if prefrontal areas are not the “true location of consciousness,” HOT may still be true [Kirkeby-Hinrup and Overgaard, (in press)].

Global workspace theory (GWT) is a cognitive theory of consciousness originally proposed by Baars (1988). Essentially, the theory suggests that information that is globally available, i.e., some cognitive content that is available to other cognitive systems, is the information that we are conscious of. This view could be interpreted as a functional theory of consciousness where consciousness is closely related to attention and working memory. Recent versions of GWT interprets “late NCCs” as the stage of processing where specific information of sensory input is amplified and re-encoded in the prefrontal–parietal network. Interestingly, the cognitive “version” of GWT could be open to the idea that insects are conscious in the same sense as mammals and humans. Some recent papers have suggested that insects have attention just like primates (Nityananda, 2016), and that bees and flies have working memory (Menzel, 2009). However, according to the “neural versions” of the same theory, suggesting that prefrontal networks are necessary for consciousness, it seems harder to defend that insects are conscious.

**Figure 1:**
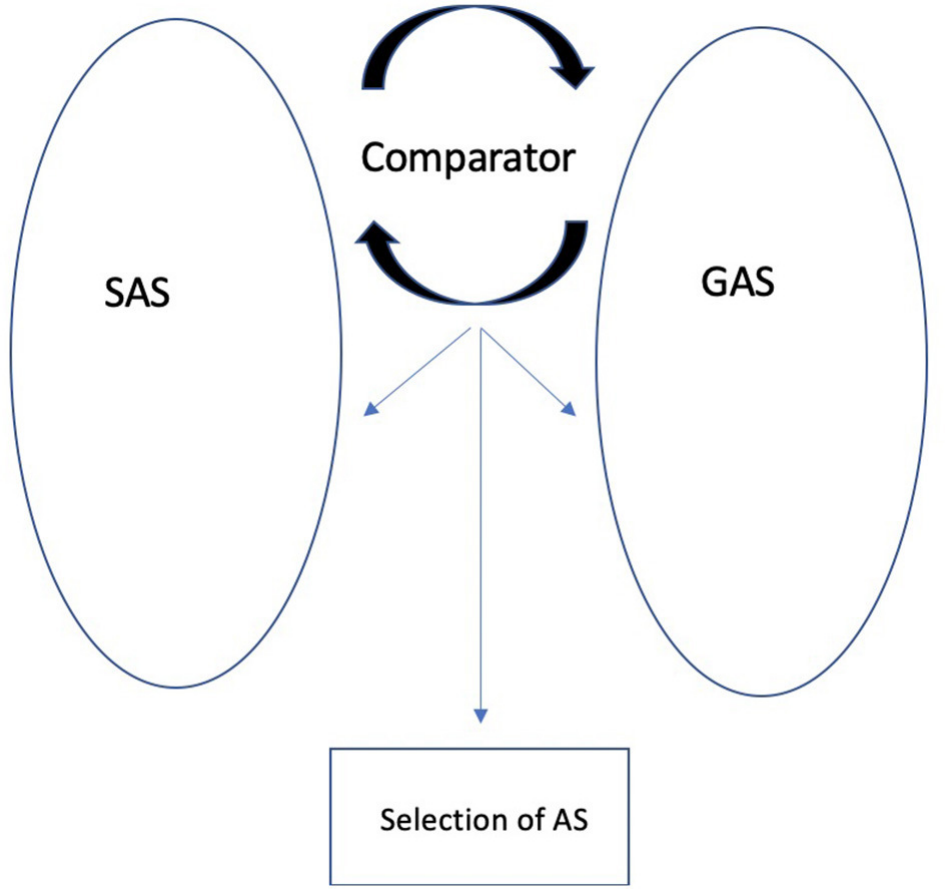
The original REF-model (re-drawn from Mogensen and Malá, 2009).

One theory that is not part of the “early” nor of the “late” camp is the REF model (see Figure 1) (e.g., Mogensen and Malá, 2009). The original REF model is focused on the mechanisms of problem solving and cognitive recovery after acquired brain injury. One of its goals is to resolve the apparent contradiction between functional localization and functional recovery. In the REF model this is done by presenting a connectionist network—within which the “units” are advanced information processing modules called Elementary Functions (EFs). Via experience-driven backpropagation processes these EFs are combined into Algorithmic Strategies (ASs). ASs are the “programs” that form the basis for task solution (in the form of overt behavior or mental representation) at the level of the Surface Phenomena. In later elaborations of the REF model, the level of Algorithmic Modules (AMs) was added (Mogensen, 2012). An AM is a combination of interacting EFs, computationally constituting a significantly higher level of information processing than what is achieved by an individual EF (Mogensen and Overgaard, 2017).

The REFCON model was presented to investigate perception and consciousness based on the same fundamental logic (e.g., Overgaard and Mogensen, 2014). The REFCON model introduces two perception specific entities: the Perceptual Elementary Function (PEF) and the Perceptual Algorithmic Module (PAM). A PEF is a specialized EF that receives a more or less direct sensory input. A PAM is a specialized AM that represents an external entity and has been organized via mechanisms similar to those of other AMs. PAMs are organized in a hierarchical manner from relatively low level PAMs—situated close to the initial sensory input—to PAMs of the highest levels. The perceptual process is initiated by the activation of a combination of PEFs, and the pattern of activated PEFs leads to activation of a number of PAMs of the lowest level. This feedforward activation is followed by a “feedback test” in which each activated PAM “interrogates” its constituent PEFs regarding their activation. In a cascade of feedforward activation followed by feedback tests, PAMs of progressively higher levels are activated. PAMs of even the highest level are, however, not in themselves the mechanism of conscious perception nor available as the basis for action. A PAM can only contribute to consciousness when it becomes integrated in a specialized AS called the Situational Algorithmic Strategy (SAS). When a high level PAM is activated it becomes more or less integrated in SAS. Under special circumstances—e.g., via top-down influences—even PAMs of lower levels may become integrated into SAS. When integrated into SAS, a PAM becomes available for cognitive access, behavioral control, and consciousness. The degree of the integration into SAS determines the level of availability.

**Figure 2:**
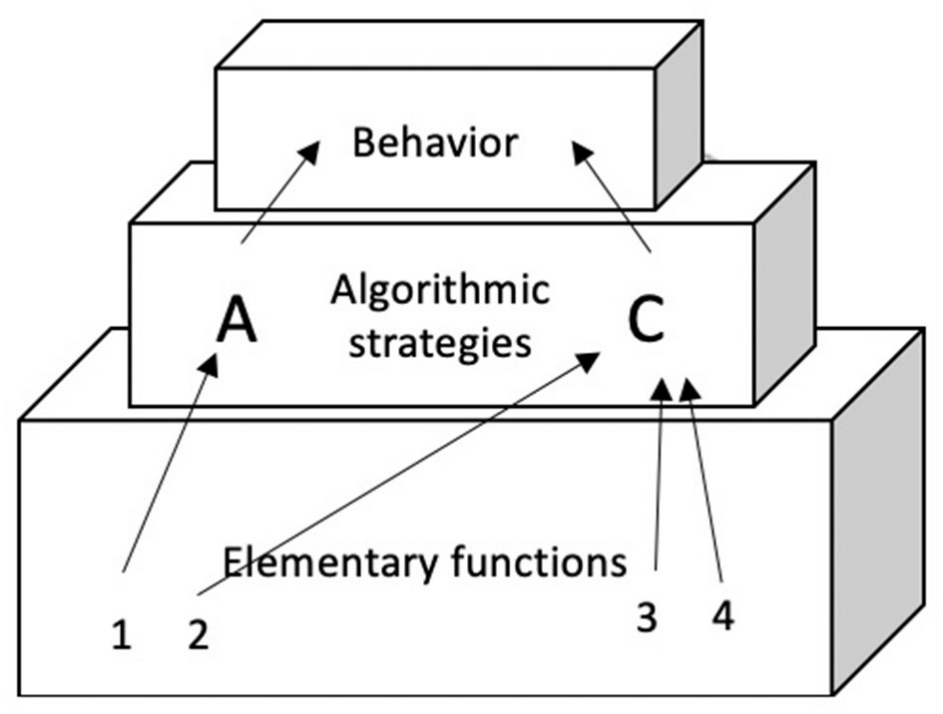
The REFGEN model (re-drawn from Mogensen and Overgaard, 2017).

The original REF model and REFCON were extended with the REFGEN model (Mogensen and Overgaard, 2017). REFGEN (see Figure 2) is based on general homeostatic principles and builds on the principles of the original REF and REFCON models. The REFGEN model introduces two new concepts: The Goal Algorithmic Strategy (GAS) and Comparator. Like SAS, GAS is a dynamic and widely distributed AS. GAS reflects the goals toward which it is desired that the individual currently moves. The Comparator constantly performs a two-way comparison between the status/structure of SAS and GAS, respectively. Via a combination of AS activation and backpropagation mechanisms, GAS works toward establishing the best possible match between SAS and GAS. The backpropagation mechanisms of GAS can modify SAS, GAS, other ASs and Comparator. Comparator will, thus, attempt to modify the structure of SAS according to the goals represented within GAS. But there will be a parallel modification of the structure of GAS as a result of what is represented within SAS.

In summary, the REF model and its extensions present the standpoint that mental states are realized in the available physical material that is continuously reorganized. The model sees the entire physical system as organized in order to realize certain actions, and the content of consciousness is the information that is available to potential action. Accordingly, there is no theoretical hindrance for consciousness to be realized in an even very differently organized neural system. This notion of availability is related to but different from the notion in Global Workspace Theory according to which information must be available to other well-defined cognitive systems.

One of the main controversies in current consciousness research is the relation between so-called phenomenal consciousness (what a subjective experience is like for a subject) and access consciousness (information that is available for use in reasoning and for direct control of action and speech) as first proposed by Block (1995). Block has consistently argued that the two concepts can be empirically dissociated (Block, 2007, 2011), others argue that the two concepts only refer to one property (Cohen and Dennett, 2011), while others again seem to argue that they are conceptually different but always empirically correlated (Chalmers, 1997).

REFCON sees consciousness and the availability to and access of information as fundamentally related (see Overgaard, 2018). Here, information available for (certain kinds of) action is conscious to some degree as the related neural activation is integrated into a neural strategy representing the current state of the individual. This has been used to explain blindsight (Overgaard and Mogensen, 2015), and the relation between so-called first and higher order states of cognition (Overgaard and Mogensen, 2017). Fazekas and Overgaard (2018) takes this idea further, and suggest concrete common mechanisms for access and phenomenal consciousness.

REF and its extensions represent a theoretical framework that is very different from the view mentioned above that structure and function are identical. The framework shows how one can conceive of multiple realization—i.e., the view that the exact same function (mental state) can be realized by different physical states or constellations hereof. Structure-function identity and multiple realization, clearly, give rise to very different answers to the question how common we should expect consciousness to be among other lifeforms.

Nothing from the REF perspective would go against the idea that species with very different brains—or potentially no brains at all—could also realize states of cognition, access, and phenomenal consciousness. In fact, this theoretical framework would very much expect insects to have conscious experience in the same sense (but most likely with different content) as human beings.

Some other recent theoretical proposals support this view that access consciousness can be seen as a guide to phenomenal consciousness. Shea and Bayne (2010) and Shea (2012) have suggested what they call “The natural kind approach” according to which we should attempt to define a cluster of non-verbal indicators which then can be used to deem non-communicating people (e.g., vegetative state patients) and other animals conscious or unconscious. The idea is related to the “facilitation hypothesis”—that conscious perception compared to unconscious perception facilitates a cluster of cognitive states related to the object of perception (Birch, 2020). Under the condition that one accepts this logic, one may use a variety of cognitive tests to investigate consciousness in insects, examining e.g., metacognition and decision making (Perry and Barron, 2013; Loukola et al., 2017), emotions (Bateson et al., 2011) or mental representations (Solvi et al., 2020). Such proposals appeal to common sense, and may be the best way forward, yet they only make sense on a theoretical background of multiple realization rather than structure-function identity.

Just as current methods and theories in consciousness set up restrictions for how to approach the topic of consciousness in insects, the existing body of research in insect cognition adds important insights to consciousness research. If one argues that consciousness should be understood as being identical to certain neural regions or processes, one would then also be forced to argue that the metacognition, decision making, emotions, and mental representations observed in insects appear in “total darkness”—without any accompanying subjective experience. Although controversial (e.g., Hassin, 2013), there is little evidence that all the above-mentioned states can occur unconsciously—as an increasing amount of experimental evidence suggests that totally unconscious cognition and perception is rare (Overgaard and Mogensen, 2015; Overgaard, 2017). This could seem as an argument in favor of multiple realization theories of consciousness.

## Conclusion

In conclusion, we have no direct evidence of consciousness in insects. Furthermore, for principle reasons, we will never be able to obtain direct measures of the presence or absence of insect consciousness. The current available theories of consciousness give very different answers to the question. According to some, it is impossible, according to others, it is in fact likely. It appears that the center of the battleground is whether specific neural substrates—observed in humans—are considered necessary for consciousness. If the answer to this question is positive, insect consciousness seems unlikely. However, if consciousness is related to certain cognitive rather than neural phenomena (as in e.g., the cognitive version of global workspace theory), insect consciousness should be possible. If consciousness is related to information available to action and is merely realized by neural substrates, but is not dependent on specific structures (as in REF), insect consciousness is in fact very likely. Based on this conclusion, it seems the question of consciousness in insects should not be dealt with directly—i.e., it seems as a hopeless errand to answer just by investigating insects themselves. However, if or when consciousness research becomes able to present answers to how we are to theoretically conceive of the general relation between subjective experience and its physical substrate, then that answer may naturally generalize to answer the question of insect consciousness also.

## Author Contributions

The author confirms being the sole contributor of this work and has approved it for publication.

## Conflict of Interest

The author declares that the research was conducted in the absence of any commercial or financial relationships that could be construed as a potential conflict of interest.

## References

[B1] Baars B. J. (1988). A Cognitive Theory of Consciousness. New York, NY: Cambridge University Press.

[B2] Barron A. B. Klein C. (2016). What insects can tell us about the origins of consciousness. PNAS 113, 4900–4908. 10.1073/pnas.1520084113 27091981 PMC4983823

[B3] Bateson M. Desire S. Gartside S.. (2011). Agitated honeybees exhibit pessimistic cognitive biases. Curr. Biol. 21, 1070–1073. 10.1016/j.cub.2011.05.017 21636277 PMC3158593

[B4] Bayne T. Hohwy J. Owen A. (2016). Are there levels of consciousness? Trends Cogn. Sci. 20, 405–413. 10.1016/j.tics.2016.03.009 27101880

[B5] Birch J. (2020). The search for invertebrate consciousness. Nous 2020, 1–21. 10.1111/nous.12351 PMC7612530 35321054

[B6] Block N. (1995). On a confusion about a function of consciousness. Behav. Brain Sci. 18, 157–162. 10.1017/S0140525X00038188

[B7] Block N. (2007). Consciousness, accessibility, and the mesh between psychology and neuroscience. Behav. Brain Sci. 30, 481–499. 10.1017/S0140525X07002786 18366828

[B8] Block N. (2011). Perceptual consciousness overflows cognitive access. Trends Cogn. Sci. 12, 567–575. 10.1016/j.tics.2011.11.001 22078929

[B9] Chalmers D. (1995). Facing up to the problem of consciousness. J. Consciousness Studies 2, 200–219.

[B10] Chalmers D. J. (1996). The Conscious Mind. New York, NY: Oxford University Press.

[B11] Chalmers D. (1997). “Availability: the cognitive basis of experience,” in The Nature of Consciousness, eds Block M. N. Flanagan O. Guzeldere G. (Cambridge: MIT Press). 421–424. 10.1017/S0140525X97240057

[B12] Cohen M. Dennett D. C. (2011). Consciousness cannot be separated from function. Trends Cogn. Sci. 15, 358–364. 10.1016/j.tics.2011.06.008 21807333

[B13] Dienes Z. Seth A. (2010). Measuring any conscious content versus measuring the relevant conscious content. Consciousn. Cogn. 19, 4, 1079–1080. 10.1016/j.concog.2010.03.009 20400336

[B14] Fazekas P. Overgaard M. (2018). A multi-factor account of degrees of awareness. Cogn. Sci. 42, 1833–1859. 10.1111/cogs.12478 28397287

[B15] Frässle S. Sommer J. Jansen A. Naber M. Einhäuser W. (2014). Binocular rivalry: Frontal activity relates to introspection and action but not to perception. J. Neurosci. 34, 5, 1738–1747. 10.1523/JNEUROSCI.4403-13.2014 24478356 PMC6827584

[B16] Fu Q. Fu X. Dienes Z. (2008). Implicit sequence learning and conscious awareness. Consciousn. Cogn. 17, 185–202. 10.1016/j.concog.2007.01.007 17383202

[B17] Hassin R. (2013). Yes it can: on the functional abilities of the human unconscious. Perspect. Psychol. Sci. 8, 195–207. 10.1177/1745691612460684 26172502

[B18] Jackson F. (1986). What Mary didn't know. J. Philos. 83, 291–295. 10.2307/2026143

[B19] Johansson P. Hall L. Sikström S. Tärning B. Lind A. (2006). How something can be said about telling more than we can know: on choice blindness and introspection. Conscious. Cogn. 15, 673–692. 10.1016/j.concog.2006.09.004 17049881

[B20] Key B. Arlinghaus R. Browman H. (2016). Insects cannot tell us anything about subjective experience or the origin of consciousness. PNAS 113:E3813. 10.1073/pnas.1606835113 27357664 PMC4941505

[B21] Kirkeby-Hinrup Overgaard M. (in press). Finding the neural correlates of consciousness will not solve all our problems. Philosoph. Mind Sci..

[B22] Lau H. Rosenthal D. (2011). Empirical support for higher-order theories of conscious awareness. Trends Cogn. Sci. 15, 365–373. 10.1016/j.tics.2011.05.009 21737339

[B23] Loukola O. Perry C. Coscos L. (2017). Bumblebees show cognitive flexibility by improving on an observed complex behavior. Science 355:834. 10.1126/science.aag2360 28232576

[B24] Menzel R. (2009). Working memory in bees – also in flies? J. Neurogen. 1–2, 92–99 10.1080/01677060802610612 19132597

[B25] Mogensen J. (2012). Cognitive Recovery and Rehabilitation After Brain Injury: Mechanisms, Challenges and Support, Brain Injury—Functional Aspects, Rehabilitation and Prevention, London:IntechOpen, 121–150. 10.5772/28242

[B26] Mogensen J. Divac I. (1982). The prefrontal “cortex” in the pigeon. Behavioral evidence. Brain Behav. Evol. 21, 60–66. 10.1159/000121617 7159828

[B27] Mogensen J. Malá H. (2009). Post-traumatic functional recovery and reorganization in animal models: a theoretical and methodological challenge. Scand. J. Psych., 561–573 10.1111/j.1467-9450.2009.00781.x 19930255

[B28] Mogensen J. Overgaard M. (2017). Reorganization of the connectivity between Elementary Functions – a model connecting conscious states to neural connections. Front. Psycho. Consciou. Res. 8, 1–21. 10.3389/fpsyg.2017.00625 PMC5397468 28473797

[B29] Mogensen J. Overgaard M. (2020). Avian prefrontal cortex and conscious experience. Science 369, 1626–1629 10.1126/science.abb1447 32973028

[B30] Nagel T. (1974). What is it like to be a bat. Philosoph. Rev. 83, 435–450. 10.2307/2183914

[B31] Nisbett R. Wilson T. (1977). Telling more than we can know: verbal reports on mental processes. Psychol. Rev. 84, 231–259. 10.1037/0033-295X.84.3.23117049881

[B32] Nityananda V. (2016). Attention-like processes in insects. Proc. R. Soc. London Series B Biol. Sci. 283:20161986. 10.1098/rspb.2016.1986 PMC5124100 27852803

[B33] Overgaard M. (2004). Confounding factors in contrastive analysis. Synthese 141, 217–231. 10.1023/B:SYNT.0000043019.64052.e0

[B34] Overgaard M. (2010). How consciousness will change our view on neuroscience Cogn. Neurosci. 1, 224–225. 10.1080/17588928.2010.497585 24168340

[B35] Overgaard M. (2017). The status and future of consciousness research. Front. Psychol. Consciousness Res. 8, 1–4. 10.3389/fpsyg.2017.01719 PMC5641373 29066988

[B36] Overgaard M. (2018). Phenomenal consciousness and cognitive access. Philosoph. Trans. R. Soc. London Series B Biol. Sci. 373:20170353. 10.1098/rstb.2017.0353 PMC6074085 30061466

[B37] Overgaard M. Fazekas P. (2016). Can no-report paradigms extract true neural correlates of consciousnesss? Trends Cogn. Sci. 20, 241–242. 10.1016/j.tics.2016.01.004 26880396

[B38] Overgaard M. Mogensen J. (2014). Conscious perception: A representational, non-reductionistic, level-dependent approach. Philosoph. Trans. R. Soc. London Series B Biol. Sci. 369:0130209. 10.1098/rstb.2013.0209 PMC3965164 24639581

[B39] Overgaard M. Mogensen J. (2015). Reconciling current approaches to blindsight. Consci. Cogn. 32, 33–40. 10.1016/j.concog.2014.08.003 25172329

[B40] Overgaard M. Mogensen J. (2017). An integrative view on consciousness and introspection. Rev. Philos. Psychol. 8, 129–141. 10.1007/s13164-016-0303-6

[B41] Overgaard M. Mogensen J. (2020). “Will we explain consciousness when we find the neural correlates of consciousness?” in Beyond Neural Correlates of Consciousness, eds Overgaard M. Kirkeby-Hinrup A. Mogensen J. (London: Routledge). 4–15. 10.4324/9781315205267

[B42] Overgaard M. Overgaard R. (2011). Measurements of consciousness in the vegetative state. Lancet 6736, 61224−61225. 10.1016/S0140-6736(11)61591-2 22078856

[B43] Perry C. Barron A. (2013). Honey bees selectively avoid difficult choices. PNAS 110, 19155–19159. 10.1073/pnas.1314571110 24191024 PMC3839751

[B44] Pitts M. Padwal J. Fennelly D. Martinez A. Hillyard S. (2014). Gamma band activity and the P3 reflect post-perceptual processes, not visual awareness. NeuroImage 101, 337–350. 10.1016/j.neuroimage.2014.07.024 25063731 PMC4169212

[B45] Ramsøy T. Overgaard M. (2004). Introspection and subliminal perception. Phenomenol. Cognit. Sci. 3, 1–23. 10.1023/B:PHEN.0000041900.30172.e8

[B46] Sandberg K. Overgaard M. (2015). “Using the perceptual awareness scale (PAS),” in Behavioral Methods in Consciousness Research, eds Overgaard M. (Oxford: Oxford University Press). 181–199. 10.1093/acprof:oso/9780199688890.003.0011

[B47] Searle J. (1990). Consciousness, explanatory inversion and cognitive science. Behav. Brain Sci. 13, 585–642. 10.1017/S0140525X00080304

[B48] Searle J. (1992). The Rediscovery of the Mind. Cambridge: MIT Press. 10.7551/mitpress/5834.001.0001

[B49] Shea N. (2012). Methodological encounters with the phenomenal kind. Philos. Phenom. Res. 84, 307–344. 10.1111/j.1933-1592.2010.00483.x PMC3361722 22654148

[B50] Shea N. Bayne T. (2010). The vegetative state and the science of consciousness. Br. J. Philoso. Sci. 61, 459–484. 10.1093/bjps/axp046 22654125 PMC3361721

[B51] Solvi C. Al-Khudhairy S. Chittka L. (2020). Bumble bees display cross-modal object recognition between visual and tactile senses. Science 367:910. 10.1126/science.aay8064 32079771

[B52] Szczepanowski R. Wierzchon M. Cleeremans A. (2013). The perception of visual emotion: comparing different measures of awareness. Conscious. Cogn. 22, 212–220 10.1016/j.concog.2012.12.003 23337441

[B53] Timmermans B. Cleeremans A. (2015). “How can we measure awareness? An overview of current methods,” in Behavioral Methods in Consciousness Research, eds Overgaard M. (Oxford: Oxford University Press). 21–49. 10.1093/acprof:oso/9780199688890.003.0003

[B54] Timmermans B. Sandberg K. Cleeremans A. Overgaard M. (2010). Partial awareness distinguishes between conscious perception and conscious content. Conscious. Cogn. 19, 1081–1083 10.1016/j.concog.2010.05.006

[B55] Tononi G. Boly M. Massimini M. Koch C. (2016). Integrated information theory: from consciousness to its physical substrate. Nat. Rev. Neurosci. 17, 450–461. 10.1038/nrn.2016.44 27225071

